# Intravenous transplantation of mesenchymal stem cells preconditioned with early phase stroke serum: current evidence and study protocol for a randomized trial

**DOI:** 10.1186/1745-6215-14-317

**Published:** 2013-10-01

**Authors:** Suk Jae Kim, Gyeong Joon Moon, Won Hyuk Chang, Yun-Hee Kim, Oh Young Bang

**Affiliations:** 1 Department of Neurology, Samsung Medical Center, Sungkyunkwan University School of Medicine, 50 Irwon-dong, 135-710, Gangnam-gu, Seoul, South Korea; 2 Clinical Research Center, Samsung Biomedical Research Institute, Seoul, South Korea; 3 Medical Research Institute, Sungkyunkwan University School of Medicine, Suwon, Gyeong-gi, South Korea; 4 Physical and Rehabilitation Medicine, Samsung Medical Center, Sungkyunkwan University School of Medicine, Seoul, South Korea

**Keywords:** Stroke, Cerebral infarction, Mesenchymal stem cells, Stem cells, Neurogenesis, Clinical trial

## Abstract

**Background:**

Recovery after a major stroke is usually limited, but cell therapy for patients with fixed neurologic deficits is emerging. Several recent clinical trials have investigated mesenchymal stem cell (MSC) therapy for patients with ischemic stroke. We previously reported the results of a controlled trial on the application of autologous MSCs in patients with ischemic stroke with a long-term follow-up of up to 5 years (the 'STem cell Application Researches and Trials In NeuroloGy’ (STARTING) study). The results from this pilot trial are challenging, but also raise important issues. In addition, there have been recent efforts to improve the safety and efficacy of MSC therapy for stroke.

**Methods and design:**

The clinical and preclinical background and the STARTING-2 study protocol are provided. The trial is a prospective, randomized, open-label, blinded-endpoint (PROBE) clinical trial. Both acute and chronic stroke patients will be selected based on clinical and radiological features and followed for 3 months after MSC treatment. The subjects will be randomized into one of two groups: (A) a MSC group (n = 40) or (B) a control group (n = 20). Autologous MSCs will be intravenously administered after *ex vivo* culture expansion with autologous ischemic serum obtained as early as possible, to enhance the therapeutic efficacy (ischemic preconditioning). Objective outcome measurements will be performed using multimodal MRI and detailed functional assessments by blinded observers.

**Discussion:**

This trial is the first to evaluate the efficacy of MSCs in patients with ischemic stroke. The results may provide better evidence for the effectiveness of MSC therapy in patients with ischemic stroke.

**Trial registration:**

This trial was registered with ClinicalTrials.gov, number NCT01716481.

## Background

Stroke is a leading cause of death, along with cancer and coronary heart disease, and the most common cause of physical disability in adults. Moreover, stroke causes a greater loss of healthy life years, as measured in disability-adjusted life years, than other illnesses [1]. Thrombolytic therapy is currently the only available stroke treatment, though it can only be applied to a limited population of patients. Various approaches to protect the brain from ischemic damage have met with limited success in clinical practice. Consequently, a large proportion of stroke survivors are left with severe disabilities.

To date, relatively little attention has been given to restorative therapy after stroke. Although rehabilitation is important for maximizing functional recovery in the early stages after stroke, no definitive treatment can repair lost brain function. Cell-based therapy is one of the most promising approaches in stroke treatment research, and has recently been evaluated as a regenerative strategy for patients with fixed neurologic deficits after stroke.

Clinical protocols must be established based on recent advances in understanding the mechanisms of stem cells in recovery after stroke. Here, we discuss the current status and important issues in the application of stem cells in ischemic stroke therapy. We also introduce the protocol and rationale of the randomized trial 'STem cell Application Researches and Trials In NeuroloGy-2’ (STARTING-2).

### Current cell-based therapy in stroke: trials and issues

Several recent clinical trials have used stem cells in stroke (Table 1) [2-9], and the results from these trials have raised important issues. Specifically, these trials varied in patient characteristics, cell therapy timing, dose and type of cells delivered, and mode of treatment. In addition, many factors that could be critical for transplantation success including the location/extension of lesions were not adequately considered [10]. Finally, the assessment of functional improvement, the occurrence of adverse effects, and pretreatment screening tests for safety were not standardized.

**Table 1:** Clinical trials of stem cell therapy in stroke patients

**Factors**	**Neural stem/progenitor cells**	**Bone marrow mononuclear cells**		**Mesenchymal stem cells**			
Lead author, year, reference	Savitz*,* 2005 [2]	Savitz*,* 2011 [4]	Friedrich, 2012 [5]	Bang*,* 2005 [6]	Lee, 2010 [7] (STARTING trial)	Honmou, 2011 [8]	Bhasin, 2011 [9]
Study design	No control group	No control group	No control group	Control, n = 25	Control, n = 36	No control group	Control, n = 6
	Treatment, N = 5	Treatment, N = 10	Treatment, N = 20,	Treatment, n = 5	Treatment, n = 16	Treatment, n = 12	Treatment, n = 6
	4 years f/u	6 months f/u	6 months f/u	1 year f/u	5 years f/u	1 year f/u	24 weeks
Brain infarct	Chronic basal ganglia infarct	Acute (24 to 72 h), large hemispheric	Acute (3 to 7 days), non-lacunar	Subacute, large cortical	Subacute, large cortical	Chronic (36 to 133 days), large cortical	Chronic (3 months to 1 year)
Cells used	Neural progenitor cells from primordial porcine striatum	Autologous bone marrow mononuclear cells		Autologous bone-marrow-derived mesenchymal stem cells			
Cell dose	2 × 10^7^ cells^a^	1 × 10^6^ cells/kg^a^	2.2 × 10^8^ cells^a^	1 × 10^8^ cells^a^	1 × 10^8^ cells^a^	1 × 10^8^ cells^a^	5 to 6 × 10^7^ cells^a^
Manipulation	Fetal porcine striatum was washed, triturated, and dissociated to yield cell suspensions	Isolation using human albumin-containing normal saline		*Ex vivo* culture expansion using fetal bovine serum		*Ex vivo* culture expansion using autologous serum	*Ex vivo* culture expansion using animal serum-free media (Stem Pro SFM)
FDA^b^	More than minimal manipulation	Minimal manipulation		More than minimal manipulation			
ICMS^c^	Early investigational cell line	Clinical grade		Clinical grade		Clinical grade	Clinical grade
Mode of application	Intralesional	Intravenous	Intraarterial	Intravenous			
Presumed mechanisms	Cell replacement and trophic support	Trophic support		Trophic support			
Efficacy	Not available	mRS 1 shift vs historical control	Good outcome (mRS 0 to 2) in 40%	Barthel index improved at 3 months	Proportion of mRS 0 to 3 increased in MSC but not control group	Improve in daily rate of NIHSS changes	Modest increase in Fugl-Meyer and mRS
Adverse effect	1 seizure, 1 worsening of weakness	None	None	None	None	None	None
Safety test	Cell viability PCR testing for porcine endogenous retrovirus	Cell viability MSC surface markers; bacteria, fungi, mycoplasma culture.	Cell viability	Cell viability MSC surface markers; bacteria, fungi, viral and mycoplasma culture.		Cell viability, MSC surface markers; bacteria, syphilis, fungi, viral, mycoplasma, endotoxin level.	Cell viability; mycoplasma, endotoxin level

^a^Equivalent to preclinical studies.

^b^US Food and Drug Administration (FDA) regulation on cell therapy.

^c^Clinical staging for cell lines by the International cellular medicine society (ICMS).

*f/u* follow-up, *mRS* modified Rankin Score, *MSC* mesenchymal stem cell, *NIHSS* National Institutes of Health Stroke Scale, *PCR* polymerase chain reaction.

#### Patient selection

Selection of candidate patients for cell-based therapies based on factors such as stroke severity, lesion location, and stroke chronicity should be optimized. Because of the experimental nature of this treatment, clinical trials of cell-based therapies for stroke have studied patients with severe disabilities or chronic stroke, sometimes several years after stroke onset. However, it may be difficult to demonstrate therapeutic benefit in these cases [11]. In contrast, patients with minor strokes might not be candidates because of the possible risks from these experimental treatments.

Most experimental stem-cell-based therapies for stroke are tested in animal models with middle cerebral artery (MCA) occlusions [12]. Stimulation of stroke-induced subventricular neurogenesis and migration of newly formed cells into adjacent ischemic areas has been suggested as one of the important mechanisms of cell therapy and is associated with functional recovery in MCA occlusion models [13]. Thus, for the criterion of lesion location, cellular therapy targeting the enhancement of neurogenesis should be applied to patients with infarctions within the MCA territory.

**Figure 1:**
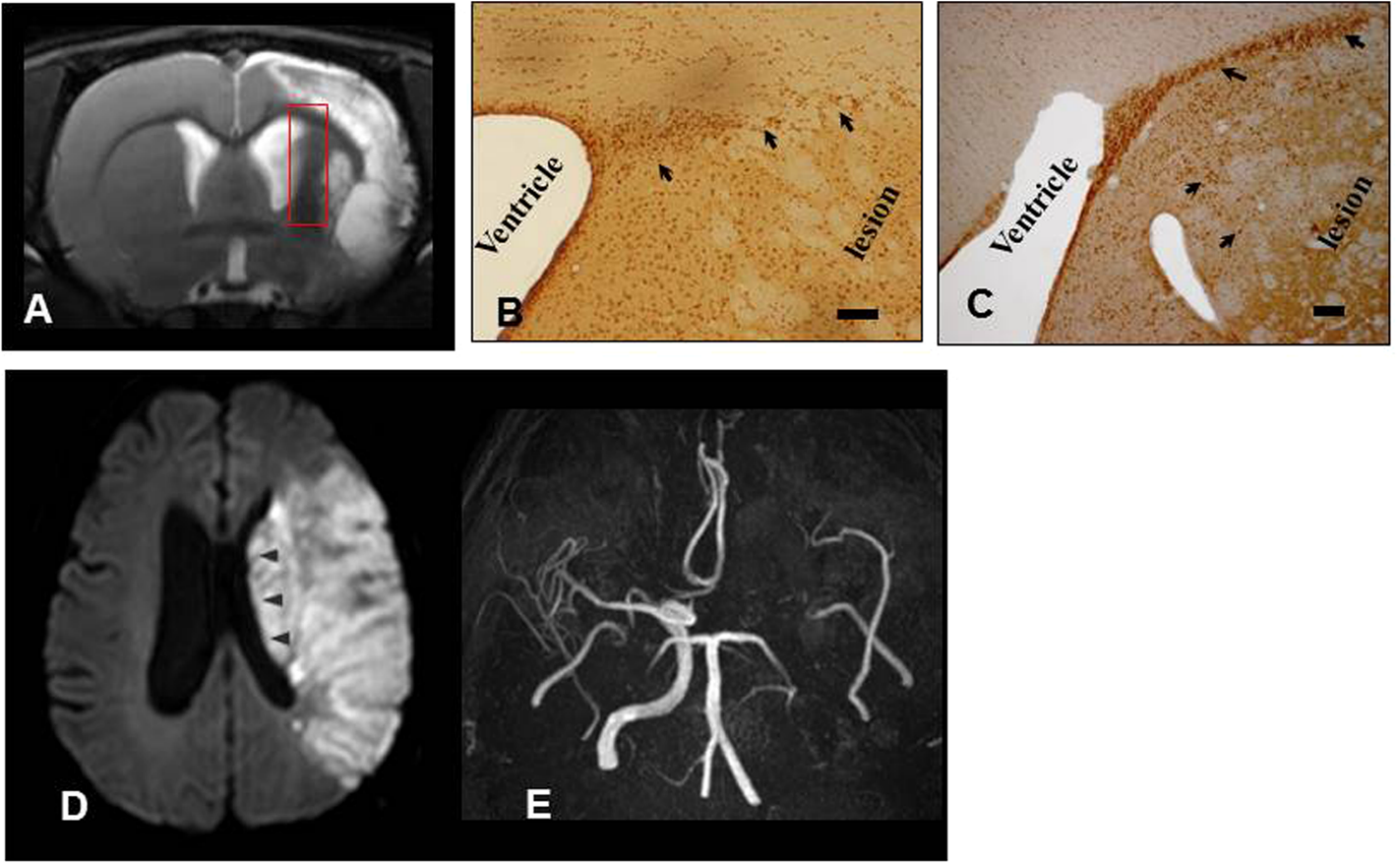
**Discrepancy between preclinical and clinical trials. (A)** MRI findings in a rat stroke model: T2-weighted MRI at 14 days after transient (90 minutes) middle cerebral artery (MCA) occlusion shows large cortical and subcortical infarcts sparing the subventricular zone (square). **(B**,**C)** Stimulated neurogenesis after application of human mesenchymal stem cells (hMSCs) in a stroke rat model: bromodeoxyuridine (BrdU) immunostaining in the subventricular zone of the ipsilateral hemisphere at day 14 showed enhancement of neurogenesis in the treated group (stroke rat that received intravenous hMSCs) **(C)** compared to placebo-treated stroke rat **(B)** (modified from Li *et al*. [18]). The arrow indicates BrdU-positive cells. Bars = 20 μm. Example of autologous hMSCs administered to a patient with stroke **(D**,**E)**. **(D)** Diffusion-weighted image demonstrates massive infarction involving most subventricular area (arrowhead). **(E)** Magnetic resonance angiography reveals persistent occlusion at the time of intravenous administration of autologous MSCs.

Preclinical studies in animal models of stroke show the importance of neurogenesis [14-16]. Newly born cells migrate to the stroke site, express neuronal and glial-specific phenotypic markers [17,18], and form synapses [19]. Transplanted stem cells might enhance the endogenous neurogenesis that occurs in certain areas, including the subventricular zone [18,20-22]. However, patients with severe stroke often have severe damage in periventricular areas, limiting endogenous neurogenesis (Figure 1). Thus, responses to cell therapy might differ depending on the degree of damage in subventricular areas [7].

Patient selection should be performed at an appropriate time [23,24]. Tissue levels of stromal cell-derivedfactor 1α (SDF-1α, also known as CXCL12, a chemoattractant of mesenchymal stem cells (MSCs)) can vary among patients with ischemic stroke. Preclinical studies have suggested that SDF-1α protein expression is associated with MSC homing and that expression is upregulated in the infarcted hemisphere for at least 1 month after stroke [25,26]. We recently showed that serum levels of SDF-1α are associated with patient response to MSC treatment [7]. The beneficial effects of cell therapy could be limited at the chronic stage, because migration and functional integration of transplanted cells with nearby neurons might be limited by scarring (gliosis) and Wallerian degeneration, as well as by decreased SDF-1α levels.

#### Optimization of treatment

The appropriate type and dose of cells, the mode of treatment, and the time of application of stem cell therapy remain to be determined [12,27]. These factors might depend on the target mechanisms of the cell therapy (that is, cell replacement vs restorative effects via trophic support) [28,29]. The target mechanisms also depend on the characteristics of the patient (for example, location and chronicity of lesions). Thus, more detailed guidelines stratified by action mechanisms are needed to provide maximal benefit to patients with different situations after a stroke [29].

Stroke lesions usually involve a variety of neuroanatomical structures that contain a diversity of cell types with complex connectivity patterns. True neuronal substitution requires specific anatomic and functional profiles [30]. This is particularly important for therapy using embryonic or induced pluripotent stem (iPS) cells. Without a functional partition, transplanted cells may even delay the recovery process or result in serious complications (that is, tumorigenesis of iPS cells transplanted in ischemic brains) [31]. Stroke causes a variety of secondary changes at locations beyond the infarct lesion. Therefore, anatomical or neurophysiologic studies (for example, diffusion-tensor imaging) evaluating the relationship of cell grafts or newly formed cells via neurogenesis to post-stroke reorganization are needed.

#### Outcome measurement

Various outcome parameters have been used in stroke clinical trials and the selection of functional endpoints has been the subject of debate. Traditional outcome measures including the National Institutes of Health Stroke Scale (NIHSS), modified Rankin score (mRS), or modified Barthel index (mBI) have advantages, but these endpoints might not be sensitive to the magnitude of changes in function expected with cellular therapy, based on the limited efficacy data from previous studies [32]. Therefore, more detailed motor assessments are needed [33]. Advanced imaging techniques might be useful for exploring action mechanisms of cell-based therapies or surrogate outcome measures [32].

#### Safety concerns

Pretreatment screening with sufficient monitoring is mandatory for safety. Unlike pharmaceutical drugs, many stem-cell-based therapies may be produced in academic laboratories where investigators are unfamiliar with the relevant regulations [34]. Recently, the US Food and Drug Administration (FDA) introduced regulations for stem-cell-based therapies [34], and many efforts have been made to avoid possible adverse events after stem cell therapy [32]. The level of regulation and oversight should be proportional to the degree of risk [35]. Minimal culture expansion is defined as an incubation period not exceeding 60 days and a number of stem cell culture passages not exceeding 10 days [36].

In addition to screening tests such as cellular viability and microbiology assays, our previous studies used flow cytometry to measure the expression of stem cell surface markers and closely monitored vascular occlusion after stem cell infusion [6,7]. Moreover, we recently reported that intra-arterial infusion of autologous MSCs causes small spotty lesions on diffusion-weighted imaging, suggesting microembolism even though no patients showed neurological deterioration [37]. MSCs are larger than mononuclear cells, prohibiting their intra-arterial application.

When stem-cell-based products require more than minimal manipulation, the cells might be grown in culture with non-human serum [34]. One of the most problematic unsolved issues in stem cell therapy is the risk of prion transmission and stimulation of immunogenicity from the use of fetal calf serum (FCS) or fetal bovine serum (FBS) in cell culture. FCS and FBS are the most widely used cell culture growth supplements, and most clinical trials use human MSCs expanded in FCS or FBS under FDA-approved protocols. However, these cells may contain potentially harmful xenogeneic components. A fluorescence microscopy study showed that FCS was not removed from cells, even after extensive washing with phosphate-buffered saline. A single injection of a common therapeutic dosage of 1 × 10^8^ MSCs grown under standard conditions would include approximately 7 to 30 mg of calf serum protein [38].

Efforts to avoid these risks have included using autologous serum or serum-free medium [39]. Recently, Honmou *et al*. used autologous MSCs culture expanded *ex vivo* with an autologous serum. They reported that the use of autologous human serum rather than FCS resulted in more rapid expansion of MSCs, which reduced cell preparation time and minimized the potential risk of transmitting viruses, prions, and proteins that can cause xenogeneic immunogenicity [8]. More recently, Bhasin and colleagues used animal serum-free media to expand MSCs in chronic stroke [9].

### Enhancing the therapeutic effects of stem cells

#### Limited efficacy of current MSC therapy strategies

The Cochrane group recently assessed the efficacy and safety of stem cell transplantation compared with conventional treatments in patients with ischemic stroke [40]. The report concluded that it is too early to know whether this intervention can improve functional outcomes and that large, well-designed trials are needed [40]. We recently reported the results of the STARTING ('STem cell Application Researches and Trials In NeuroloGy’) study, a randomized controlled trial of autologous MSC transplantation in patients with subacute severe stroke [7]. Intravenous autologous administration of MSCs cultured in FBS-containing medium was safe for patients with stroke over approximately 5 years. However, many patients remained significantly disabled, although the proportion with mRS 0 to 3 significantly increased in the MSC group but not in the control group. Thus, further trials with efforts to enhance the therapeutic effects of stem cells are needed.

#### Improving efficacy of MSC therapy and ischemic preconditioning

Efforts to improve the efficacy of MSCs include ischemic preconditioning [41,42], blood–brain barrier (BBB) manipulation [43], and use of genetically modified MSCs (although this is not feasible in clinical practice) [44,45]. Ischemic preconditioning enhances ischemic tolerance in various tissues including heart and brain [46,47]. Clinical and preclinical studies of cerebral ischemia demonstrated that brief, non-lethal ischemia and reperfusion, referred to as ischemic preconditioning, can have a protective effect against further episodes of brain ischemia [46,48]. The cytoprotective effect of preconditioning also enhances the survival of transplanted stem cells [49]. Ischemic preconditioning before transplantation of the donor cells might initiate survival signaling, creating a primed and activated state in these cells, and reinforcing their ability to withstand harsh microenvironments after transplantation [50]. Hypoxic preconditioning is used in most preclinical studies, with stem cells exposed to 0.5% to 3% oxygen for 24 to 72 h.

The number of clinical trials using allogeneic-based cell therapy approaches is growing [51]. However, treatment using bone marrow stromal cells from stroke rats was found to promote more improvement of functional outcomes in a rat stroke model compared to cells from normal rats [52]. Thus, it is conceivable that MSCs from patients with acute stroke could have different characteristics from cells from healthy individuals (either allogeneic or chronic stroke donors), and culture expansion using serum obtained during the acute phase of stroke could improve the therapeutic effects of MSCs. We recently conducted preclinical studies on the effects of ischemic preconditioning with ischemic serum on MSC functions (unpublished data). We evaluated the characteristics of rat MSCs after culturing with FBS or serum obtained from a rat stroke model. Compared to FBS, the use of serum from the rat stroke model resulted in more rapid MSC expansion, which reduced the cell preparation time by increasing the G2/M phase, decreasing cell death/senescence, increasing trophic factor secretion, and migration capacity.

An important issue in improving the therapeutic effects of cell therapy in stroke is BBB manipulation. Systemically administered cells might not need to enter the brain to have therapeutic effects but might be able to act in the periphery to increase the trophic support that enhances endogenous repair mechanisms [53]. However, even if this is the case, BBB manipulation might be needed to allow central nervous system entry of endogenous or graft-derived trophic factors [43].

## Methods and design

### The STARTING-2 trial

#### Trial characteristics and design

STARTING-2 is the first study to evaluate the efficacy of MSC treatment in patients with ischemic stroke. The study is a prospective, randomized, open-label, blinded-endpoint (PROBE) clinical trial. Both acute and chronic cases of stroke will be included, and followed for 3 months after MSC treatment. The ratio of MSC-treated to control patients is 2:1. Patients will intravenously receive autologous MSCs after *ex vivo* culture expansion with autologous ischemic serum obtained as early as possible to enhance the therapeutic efficacy (ischemic preconditioning). Selection of patients will be based on clinical and radiological features, excluding patients with large involvement of periventricular regions. BBB manipulation using intravenous mannitol before MSC treatment and comprehensive and objective measurements using multimodal MRI and detailed functional assessments will be performed.

#### Study objectives

The study tests the hypothesis that patients with ischemic stroke with moderate to severe persistent neurologic deficits will have better outcomes with intravenous transplantation of autologous MSCs expanded with autologous serum obtained during the acute phase of stroke than patients receiving standard treatment.

We chose a categorical shift in mRS at 90 days after treatment as the primary endpoint. The mRS is the primary endpoint of most stroke clinical trials and shift analysis has advantages over the classical dichotomized method for interventions that confer a uniform and modest benefit to patients over a broad range of stroke severity [54,55].

The secondary objectives are: (a) to determine the efficacy of MSC therapy by serial assessment of detailed motor function and by comparing the functional outcome between MSC therapy and control groups at 90 days after treatment; (b) to determine the safety of MSC therapy in patients with ischemic stroke; and (c) to evaluate blood and imaging biomarkers that influence the effects of MSC therapy.

#### Patient population and evaluation

**Figure 2:**
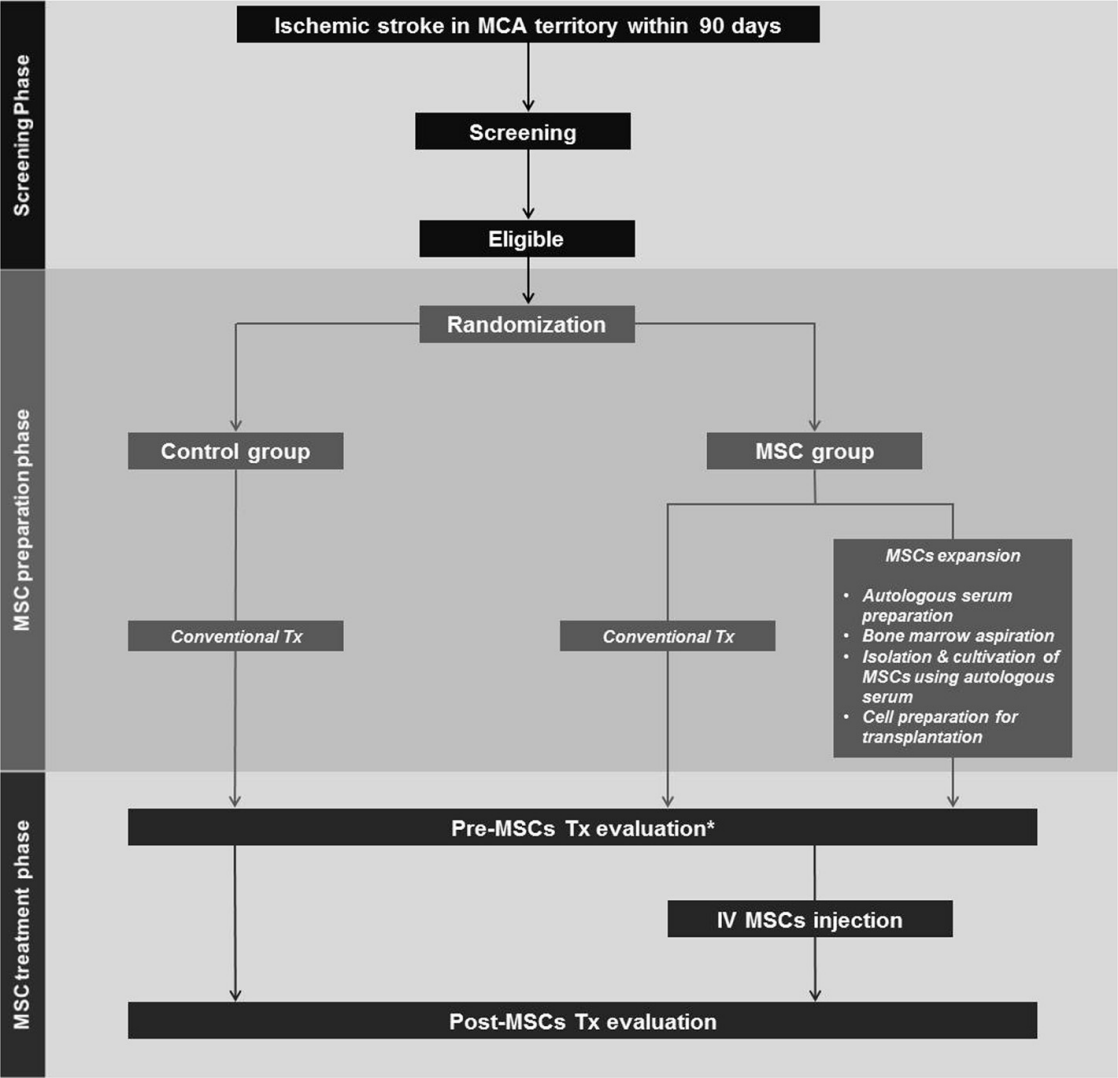
**Overall study flow of the 'STem cell Application Researches and Trials In NeuroloGy-2’ (STARTING-2) study.** *Indicates 1 day before mesenchymal stem cell (MSC) infusion in the MSC group vs 30 days (± 2 days) after randomization in the control group.

**Figure 3:**
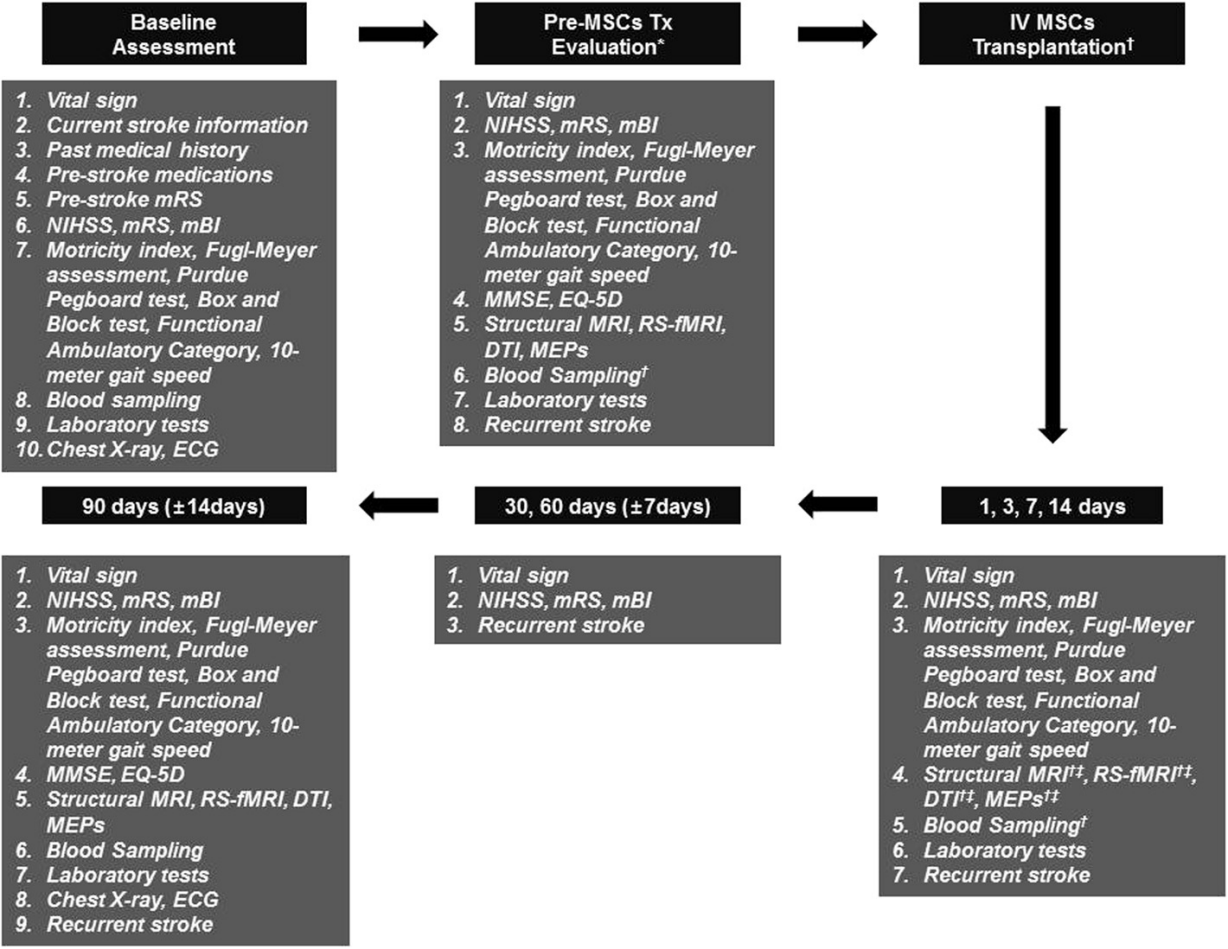
**Study protocol of 'STem cell Application Researches and Trials In NeuroloGy-2’ (STARTING-2) study at each timepoint.** *Indicates 1 day before mesenchymal stem cell (MSC) infusion in the MSC group vs 30 days (± 2 days) after randomization in the control group. †MSC group only. ‡Will be performed at 14 days after MSC transplantation.

The clinical trial protocol and consent form were approved by the Korean FDA and the Institutional Review Board of the Samsung Medical Center. Written informed consent will be obtained from all patients and/or their first-degree relatives. Inclusion and exclusion criteria are shown in Table 2. During the inpatient rehabilitation period, all participants will receive conventional rehabilitation therapy (physical, occupational, speech/language, or cognitive rehabilitation therapy as needed). Investigators blinded to treatment allocation will measure neurological disability using the NIHSS and functional scores for mRS and mBI. Motor parameters will be evaluated by the Motricity index and Fugl-Meyer assessment for gross motor function, Purdue pegboard test and box and block test for fine motor function, and functional ambulatory category and 10-m gait speed tests for mobility, for further demonstration and characterization of motor recovery [56-60]. Cognition (Mini-Mental Status Examination (MMSE)) and quality of life (EuroQol Five Dimensions (EQ-5D) questionnaire) tests will also be administered. Any complications or adverse effects observed during the study period will be described in detail. Flow diagrams of the study protocol are shown in Figures 2 and 3.

**Table 2:** Inclusion and exclusion criteria

**No.**	**Criteria type**
Inclusion	
1	Men or women (women must be of non-child-bearing potential), age 30 to 75 years
2	Stroke observed within 90 days of the onset of symptoms
3	Radiological:
	Relevant lesions within the MCA territory as assessed using DWI.
	Maximum diameter of the stroke region in any dimension ≥15 mm
	Damage not involving more than a half of the ipsilateral subventricular zone^a^
4	Clinical (National Institutes of Health Stroke Scale (NIHSS)):
	a. Moderate to severe persistent neurologic deficit (NIHSS of 6 to 21 inclusive)
	b. New onset of extremity paresis on the affected side, defined as a score of 2 to 4 on the NIHSS motor arm (item 5) or leg (item 6) question.
	c. Alert or drowsy but easily arousable as defined by score of 0 to 1 on the NIHSS level of consciousness question (item 1A).
	d. 'Slow recovery’ defined as change in NIHSS ≤1 point/3 days
5	Willingness:
	a. Reasonable likelihood of receiving standard physical, occupational and speech rehabilitation therapy as indicated for post-stroke deficits
	b. Able to participate in the evaluation process to the point of accurate assessment
	c. Willing and able to comply with scheduled visits, lifestyle guidelines, treatment plan, laboratory tests, and other study procedures
	d. Evidence of a personally signed and dated informed consent document
6	Use of antiplatelet, anticoagulant and/or antithrombotic agents is acceptable
Exclusion	
1	Presence of significant disability prior to the current stroke, defined as pre-stroke modified Rankin score of 2 or more
2	Stroke that is either:
	a. Lacunar infarction
	b. Hematologic cause of stroke
	c. Recurrent or progressive stroke within 1 week at the time of screening
3	Hematologic disorders or bone marrow suppression
4	Severe medical illness defined as:
	a. Severe heart failure
	b. Severe febrile illness
	c. Hepatic or renal dysfunction
	d. Active cancer
	e. Any evidence of chronic comorbid condition or unstable acute systemic illnesses which, in the investigator’s opinion, could shorten survival or limit ability to complete the study
5	Presence of HIV, HBV, HCV, or syphilis on admission blood tests
6	Presence of active depression not adequately controlled that interferes with major activities of daily living immediately prior to the current stroke
7	Presence of dementia prior to the current stroke that is likely to confound clinical evaluation
8	Lactating women or pregnant women as determined by positive urine hCG test
9	Considered unwilling or unable to comply with the procedures and study visit schedule outlined in the protocol
10	Unwilling to undergo bone marrow aspiration

^a^We will measure the degree of involvement of the ipsilateral subventricular zone on the initial diffusion-weighted image at two axial levels; (a) the upper thalamus and head of caudate nucleus and (b) the corona radiate (7-mm upper level).

*DWI* diffusion-weighted image, *HBV* hepatitis B virus, *HCV* hepatitis C virus, *hCG* human chorionic gonadotropin, *MCA* middle cerebral artery.

Multimodal brain MRI including structural MRI, resting-state functional MRI (RS-fMRI) and diffusion tensor imaging (DTI) and motor evoked potentials (MEPs) will be performed. Multimodal MRI and MEPs techniques are described in the Additional file 1. Exploration of biomarkers is planned to further investigate the mechanisms of action of MSCs and to find predictors for response to cell therapy. Biomarkers will include SDF-1α (a chemokine), S100β (a marker of protection and regeneration), hypoxia-inducible factor 1α (HIF-1α, a marker of preconditioning), circulating MSCs and MSC-derived microvesicles, brain-derived neurotrophic factor (BDNF) and vascular endothelial growth factor (VEGF) levels, and BDNF genetic polymorphisms (Table 3) [7,18,61-64].

**Table 3:** Study endpoints

**Objective**	**Endpoint**
Primary endpoint of efficacy:	
Functional outcome	Categorical shift in mRS at 90 days after treatment
Secondary endpoints of efficacy:	
Disability	Change in NIHSS between pretreatment and 90 days post-treatment ≥5 points improvement or score of 0 to 2 on NIHSS score at 14 days after treatment
Functional outcome	mRS ≤2 at 90 days after treatment
	Change of mRS between pretreatment and 90 days post-treatment
	mBI ≥60 at 90 days after treatment
	Change of mBI between pretreatment and 90 days post-treatment
Further demonstration and characteristics of motor recovery	Change of gross motor function between pretreatment and 90 days post-treatment
	Motricity index and Fugl-Meyer assessment (upper, lower)
	Change of fine motor function between pretreatment and 90 days post-treatment
	Purdue pegboard test (simple) and box and block test
	Change of mobility between pretreatment and 90 days post-treatment
	Functional ambulatory category and 10-m gait speed
Cognition	Change of MMSE between pretreatment and 90 days post-treatment
Quality of life	Change of EQ-5D between pretreatment and 90 days post-treatment
Secondary endpoints of safety:	
Death	All causes of death
Recurrence	Recurrent stroke or transient ischemic attack
The immediate reaction	Allergic reactions (tachycardia, fever, skin eruption, leukocytosis)
	Local complications (hematoma or local infection at the site of bone marrow aspiration)
	Vascular obstruction (tachypnea, oliguria, or peripheral vascular insufficiency)
	Systemic complications (infections, AST/ALT, or BUN/Cr levels)
Long-term adverse effects possibly related to MSC treatment	Tumor formation (physical examination, plain X ray, f/u MRI at 90 days after treatment)
	Aberrant connections (newly diagnosed seizure or arrhythmia)
Other parameters related to efficacy:	
Exploration of biomarkers to further demonstrate the mechanism of action and genetic profile	SDF-1α (chemokine)
	S100β (protection and regeneration)
	HIF-1 (preconditioning)
	Circulating MSCs and MSC-derived microparticles
	BDNF levels and polymorphisms and VEGF levels
Multimodal MRI	Resting-state functional MRI and diffusion tensor imaging
Neurophysiologic study	Motor evoked potentials

*ALT* alanine aminotransferase, *AST* aspartate aminotransferase, *BDNF* brain-derived neurotrophic factor, *BUN* blood urea nitrogen, *Cr* creatinine, *DWI* diffusion-weighted image, *EQ-5D* EuroQol Five Dimensions, *HBV* hepatitis B virus, *hCG* human chorionic gonadotropin, *HCV* hepatitis C virus, *HIF* hypoxia-inducible factor, *mBI* modified Barthel index, *MCA* middle cerebral artery, *MMSE* Mini-Mental State Examination, *mRS* modified Rankin Score, *MSC* mesenchymal stem cell, *NIHSS* National Institutes of Health Stroke Scale, *SDF-1α*, stromal cell-derived factor 1α, *VEGF* vascular endothelial growth factor.

#### Preparation and transplantation of MSCs

Methods for bone marrow aspiration, MSC isolation, cell preparation, and intravenous infusion will be as previously described, except for the amount of aspirated bone marrow and the use of autologous serum for *ex vivo* cultivation of MSCs [6,7]. Briefly, 60 mL of bone marrow will be aspirated from both posterior iliac crests of each patient in the MSC group. Aspiration will be performed within 1 week after randomization to the MSC group. Bone marrow mononuclear cells will be separated by Ficoll density centrifugation.

We will use autologous MSCs prepared with autologous serum, instead of FBS. Serum for ischemic preconditioning will be obtained at the earliest time possible, immediately after randomization. Less than 500 mL of whole blood will be drawn at any one time.

Mononuclear cells will be cultured in a 175 cm^2^ flask (Falcon, Franklin Lakes, NJ, USA) in low-glucose Dulbecco’s modified Eagle medium (Gibco-BRL, Grand Island, NY, USA) supplemented with 10% autologous serum and 20 μg/mL gentamicin in a humidified incubator at 37°C under 5% CO_2_. Non-adherent cells will be removed when the medium is exchanged on days 5 to 7. When primary MSCs reach 70% to 80% confluence, they will be harvested and subcultured. Autologous MSCs will be culture expanded to 1 × 10^6^ cells/kg, the human dose equivalent to doses found to be effective in a rat stroke model (1 × 10^5^ to 3 × 10^6^ cells/rat) based on mean body weight [6]. Immediately before MSC infusion, 100 mL of 20% mannitol will be injected intravenously to open the BBB [43]. Expanded autologous MSCs at 1 × 10^6^ cells/kg (maximum 1.2 × 10^8^) will be transplanted through the antecubital vein with 5 × 10^6^ cells/mL of normal saline over 10 minutes. We will use good manufacturing practice conditions (Pharmicell Corporation, Seongnam, South Korea) and clinical grade reagents for cell preparation.

Cell viability will be determined by trypan blue staining at the end of harvest and before infusion, whether the viability is greater than 95%. Cell cultures will be tested weekly for bacterial, fungal, viral, or mycoplasmal contamination. Since stem cells are highly likely to be differentiated, the surface expression of CD105, CD90, CD73 (positive MSC markers), and CD34 (a negative MSC marker) will be measured on culture-expanded MSCs using flow cytometry (FACScan; Becton-Dickinson, Rutherford, NJ, USA) before intravenous transplantation into each patient. Each MSC harvest is expected to yield a homogenous population of cells with high expression of positive markers (>90% of cells) and low expression of the negative marker (<1% of cells).

#### Randomization and outcomes

Randomization will be in a 2:1 ratio of MSC-treatment to control patients using computer-generated random-permuted blocks with blocks of six subjects. The 2:1 ratio was selected to obtain a sufficient number of participants for explorative analysis in the MSC group. For the primary outcome analysis, the categorical shift in mRS at 90 days after treatment will be determined as 0 to 5 mRS levels. Deaths (a mRS score of 6) will be included in the category of worst outcome (a mRS score of 5). Secondary and exploratory outcomes are shown in Table 3.

#### Sample size and statistical analysis

Approximately 60 participants (MSCs vs control group = 40:20) will be studied. This sample size was chosen to provide a power of 80% and an alpha level of 5% to detect a common odds ratio of 4.75 (across all cut-off points of the mRS), which was the result from the STARTING trial [7]. Possible harmful effects of MSCs will be determined and assumptions underlying the sample size calculation will be adjusted by an interim analysis performed by an independent data and safety monitoring committee (data from 20 eligible participants in the MSC group) [65].

All randomized participants will be included in the endpoint analyses on an intention-to-treat basis. For primary outcome analysis, the categorical shift of mRS at 90 days after treatment will be compared between the MSC and control groups using the Cochran-Mantel-Haenszel shift test and proportional odds logistic regression analysis adjusted for age, sex, stroke mechanisms, and infarct volume on fluid attenuation inversion recovery (FLAIR) imaging 1 day before MSC infusion in the MSC group or 30 days (± 2 days) after randomization in the control group. In this study, the adjusted result is prespecified as the primary outcome analysis. Secondary and exploratory analyses will be performed according to standard statistical methods as appropriate.

## Discussion

Preclinical and clinical trials have great potential to improve the therapeutic efficacy and safety of MSCs. In the STARTING-2 trial, we are incorporating ischemic preconditioning using ischemic serum, BBB manipulation, and strict selection of candidates for stem cell therapy to improve the therapeutic effects and safety of MSCs. We anticipate that the study results may provide better evidence for the effectiveness of MSC therapy in patients with ischemic stroke.

## Trial status

Start date: November 2012.

Expected end date: February 2016.

Expected publication date: May 2016.

Status at time of submission of this article: recruitment ongoing.

## Competing interests

The authors have no competing interests to report.

## Authors’ contributions

The idea behind the study was mainly conceived by OYB. All authors participated in the study concept and design. OYB is the grant holder. SJK and OYB drafted the manuscript and will carry out the study primarily. GJM, WHC, and Y-HK contributed to writing the manuscript. All authors contributed to the execution of the study, and approved the final draft.

## Supplementary Material

Additional file 1 Methods for multimodal MRI. Click here for file
