# Supplementary material for: Intravenous transplantation of mesenchymal stem cells preconditioned with early phase stroke serum: current evidence and study protocol for a randomized trial
Source: Trials. 2013 Oct 1;14:317. doi: 10.1186/1745-6215-14-317 (PMC4016561; doi:10.1186/1745-6215-14-317)
Supplement: Additional file 1 — Methods for multimodal MRI. [file 1745-6215-14-317-S1.doc]

**Supplementary material**

**Methods for multimodal MRI**

Multimodal brain mapping will include structural MRI, resting-state functional MRI (RS-fMRI), and diffusion tensor imaging (DTI) using a Philips ACHIEVA MR scanner (Philips Medical Systems, Best, The Netherlands) operating at 3 Tesla. A total of 46 diffusion-weighted images will be obtained using the single-shot echo-planar imaging (EPI) sequence (number of slices = 60, slice thickness = 2.25 mm, matrix size = 112 × 112, in-plane resolution = 1.96 mm × 1.96 mm). The 46 images will consist of a single image acquired without diffusion gradients and 45 images acquired with diffusion gradients along respective directions. A high-resolution T1-weighted structural image will be acquired using a 3D gradient echo sequence (repetition time [TR] = 13.91 ms, echo time [TE] = 6.89 ms, number of slices = 124 slices, slice thickness = 1.6 mm, matrix size = 512 × 512, in-plane resolution = 0.47 mm × 0.47 mm). We will evaluate fiber connectivity using fiber assignment by continuous tracking (FACT), a 3D-fiber reconstruction algorithm in Philips PRIDE software. The termination criteria used for fiber tracking will be FA < 0.2 and angle change > 70◦. We will use the 3-region-of-interest (ROI) method to find the corticospinal tract (CST). The ROI will include each motor cortex, each upper anterior pons and each lower anterior pons. Fibers leading to the cerebellum will be excluded.

In addition, RS-fMRI data will be acquired. During the resting-state, subjects were instructed to keep their eyes closed and remain motionless. At each session, a total of 100 whole-brain images were collected using a T2*-weighted gradient echo EPI sequence (repetition time (TR)=3,000 msec, echo time (TE)=35 msec, number of slices=35, slice thickness=4 mm, matrix size=128×128, field of view=220 mm×220 mm). fMRI data will be preprocessed using SPM8 (Wellcome Institute of Cognitive Neurology, London, UK) and AFNI software (Scientific and Statistical Computing Core, National Institute of Mental Health, Bethesda, MD, US). Preprocessing steps will include spatial realignment to the mean volume of a series of images, normalization into the same coordinate frame as the MNI-template brain, band-pass filtering between 0.01-0.08 Hz, and smoothing using a Gaussian filter of 8 mm FWHM.

Finally, we will investigate the relationship between the degree of damage to the subventricular zone and the degree of improvement in both the control and MSC groups during the follow-up period, as previously described.[7] Briefly, we will measure the degree of involvement of the ipsilateral subventricular zone on the initial diffusion-weighted image at two axial levels; (a) the upper thalamus and head of caudate nucleus and (b) the corona radiate (7 mm-upper level).

**Methods for motor evoked potentials**

Motor evoked potentials (MEPs) will be obtained using Rapid® II stimulator (Magstim Co. Ltd., UK) with a 70-mm figure of eight-shaped coil and electromyographic recording apparatus (Synergy, Medelec Co. Ltd, UK). MEPs will be recorded from each hand using surface electrodes attached over the patient’s first dorsal interosseus (DI) muscle. A cotton cap with marks 1 cm apart will be applied to the scalp of patient. In order to obtain more highly discriminative results, we will make the target muscles relaxed. The hot spot will be defined as the optimal scalp position where reproducible muscle response will be as elicited with the lowest stimulation intensity. Motor threshold will be defined as the minimum stimulation intensity that will produce at least three MEPs exceeding 50 uV in 6 trials at the hot spot. Amplitude and latency will be measured from MEPs elicited from stimulations over the hot spot at 120% of the motor threshold.
